# Apical-out polarity in epithelial spheroids requires α6β4 integrins, cell proliferation and anchorage independence

**DOI:** 10.1242/jcs.264323

**Published:** 2026-07-13

**Authors:** Niharika Patel, Jenna Pirrello, Skylar Lynch, Hailee Patel, Bradley J. Sweeney, Shivam Priya, Tanmay P. Lele, Aki Manninen, Daniel E. Conway

**Affiliations:** ^1^ Department of Biomedical Engineering, The Ohio State University, Columbus, Ohio 43210, USA; ^2^ Department of Biomedical Engineering, Texas A&M University, College Station, Texas 77843, USA; ^3^ Department of Cancer Biology and Genetics, College of Medicine, The Ohio State University, Columbus, Ohio 43210, USA; ^4^ Artie McFerrin Department of Chemical Engineering, Texas A&M University, College Station, TX 77843, USA; ^5^ Department of Translational Medical Sciences, Texas A&M Institute of Biosciences & Technology; Houston, TX, 77030, USA; ^6^ Texas A&M University School of Engineering Medicine, Houston, TX, 77030, USA; ^7^ Disease Networks Research Unit, Faculty of Biochemistry and Molecular Medicine, Biocenter Oulu, University of Oulu, Oulu 90220, Finland; ^8^ The Ohio State University and Arthur G. James Comprehensive Cancer Center, Columbus, Ohio 43210, USA

**Keywords:** Apical-basal polarity, Integrins, Anchorage independence

## Abstract

Epithelial cells primarily segregate transmembrane proteins to apical or basal surfaces, establishing apical-basal polarity. In 3D tissues, apical proteins face inwards. Recent work by our group has shown that increased RhoA activation causes epithelial spheroids to invert apical-basal polarity via a collective rearrangement of cells, a process we and others have termed eversion. In this work, we determined that α6β4-integrin–laminin interactions are required for spheroids to evert to apical-out polarity. Additionally, we show that increased cell proliferation and anchorage independence are required to sustain apical-out polarity. We also observed that apical-out spheroids can ‘revert’ to apical-in polarity through apoptotic cavitation of cells located in the center of the spheroids. This study provides new mechanistic insights into the biochemical and biophysical mechanisms that drive eversion and maintain apical-out polarity, and supports the concept that apical-basal polarity orientation might drive phenotypic switching of epithelia.

## Teaser

**Summary:** Epithelial spheroids require α6β4 integrin–laminin interactions to transition from apical-in to apical-out polarity. Apical-out polarity is sustained through increased cell proliferation and anchorage independence.

## INTRODUCTION

Establishment and maintenance of apical-basal polarity is a crucial function for epithelia that develops and maintains epithelial tissue organization (Rodriguez-Boulan and Macara, 2014). This involves the segregation of transmembrane proteins into distinct vesicles at the trans-Golgi network for trafficking to either the apical or basal sides of the cell (Stoops and Caplan, 2014). In ductal, glandular or tubular epithelial tissue structures apical-basal polarity is established such that the apical membrane surface faces inwards (apical-in) towards a liquid-filled lumen structure. The formation and maintenance of the lumen structure is driven by establishment of apical-basal polarity. Apical actin polymerization has been shown to drive the initial formation of a lumen (Indana et al., 2024). Following the establishment of barrier function through tight junction formation, apical ion secretion establishes an osmotic pressure that mechanically stabilizes the lumen (Bovyn and Haas, 2024; Indana et al., 2024; Narayanan et al., 2020). In addition to the role of apical actin polymerization and osmotic pressure for driving lumen formation, under certain conditions, apoptotic cavitation has also been shown to be important factor for the formation of lumens (Martín-Belmonte et al., 2008).

Loss of apical-basal polarity can drive tissue disorganization and is a hallmark of carcinoma (cancers originating from epithelia) (Royer and Lu, 2011). In addition to a complete disruption of apical-basal polarity during cancer progression, it is also possible for apical-basal polarity to become ‘inverted’ where apical and basal transmembrane proteins remain segregated but instead are organized such that the apical surface faces outwards (apical-out). Apical-out polarity is a defining characteristic of specific epithelial cancers, including micropapillary carcinomas, and is associated with increased invasion (Nassar, 2004). Mostov and colleagues have also shown that apical-out polarity can be established in non-cancerous epithelial spheroids by various stimuli that alter apical-basal protein trafficking (O'Brien et al., 2001; Yu et al., 2007).

Recently our group and others have shown that both non-cancerous and cancerous epithelial spheroids can transition from normal, apical-in polarity to apical-out polarity through a collective rearrangement of cells that flip the spheroid inside-out (Narayanan et al., 2023), a process that we and others have termed eversion (Co et al., 2019, 2021; Narayanan et al., 2023). Our group developed a biophysical model to explain eversion, in which differences in the apical and basal surface energies provides a driving force for eversion (Narayanan et al., 2023). While others observed eversion when spheroids are removed from the extracellular matrix (ECM) (Co et al., 2019, 2021), a notable aspect of our prior work was that we were able to observe eversion occur within ECM (Narayanan et al., 2023), which suggests the possibility that eversion could occur in tissues under physiological or pathological conditions. Despite our biophysical model for how mechanical forces promote eversion, there is still an incomplete understating of the biochemical mechanisms that enable eversion and sustain apical-out polarity.

Given the collective movement of cells during eversion, we speculated that specific integrin–ECM interactions might be necessary for eversion to occur. By screening the response of integrin knockout MDCK cell lines to an activator of Rho, we determined that cells lacking α6 or β4 integrin had significantly reduced incidence of eversion, even though these cells exhibited lumen collapse in response to Rho activation. Integrin α6-blocking antibodies also inhibited RhoA-driven eversion in both MDCK and Caco-2 cells. Additionally, MDCK spheroids cultured in collagen failed to assemble a basal lamin–ECM network and did not evert in response to RhoA activation. These data indicate that α6β4-integrin–laminin interactions are likely necessary for enabling the collective movement of cells required to invert apical-basal polarity through the process of eversion. We also show that everted, apical-out spheroids can return to apical-in polarity when RhoA activator is removed by undergoing apoptotic cavitation. Both increased proliferation and anchorage independence are required for maintaining apical-out polarity, as inhibitors of DNA synthesis and focal adhesion kinase activity prevent eversion and restore normal apical-basal polarity. In summary, our study provides new mechanistic insights into the biochemical and biophysical mechanisms that drive eversion and maintain apical-out polarity.

## RESULTS

### Involvement of α6β4 integrins and laminin ECM in eversion

**Fig. 1.**
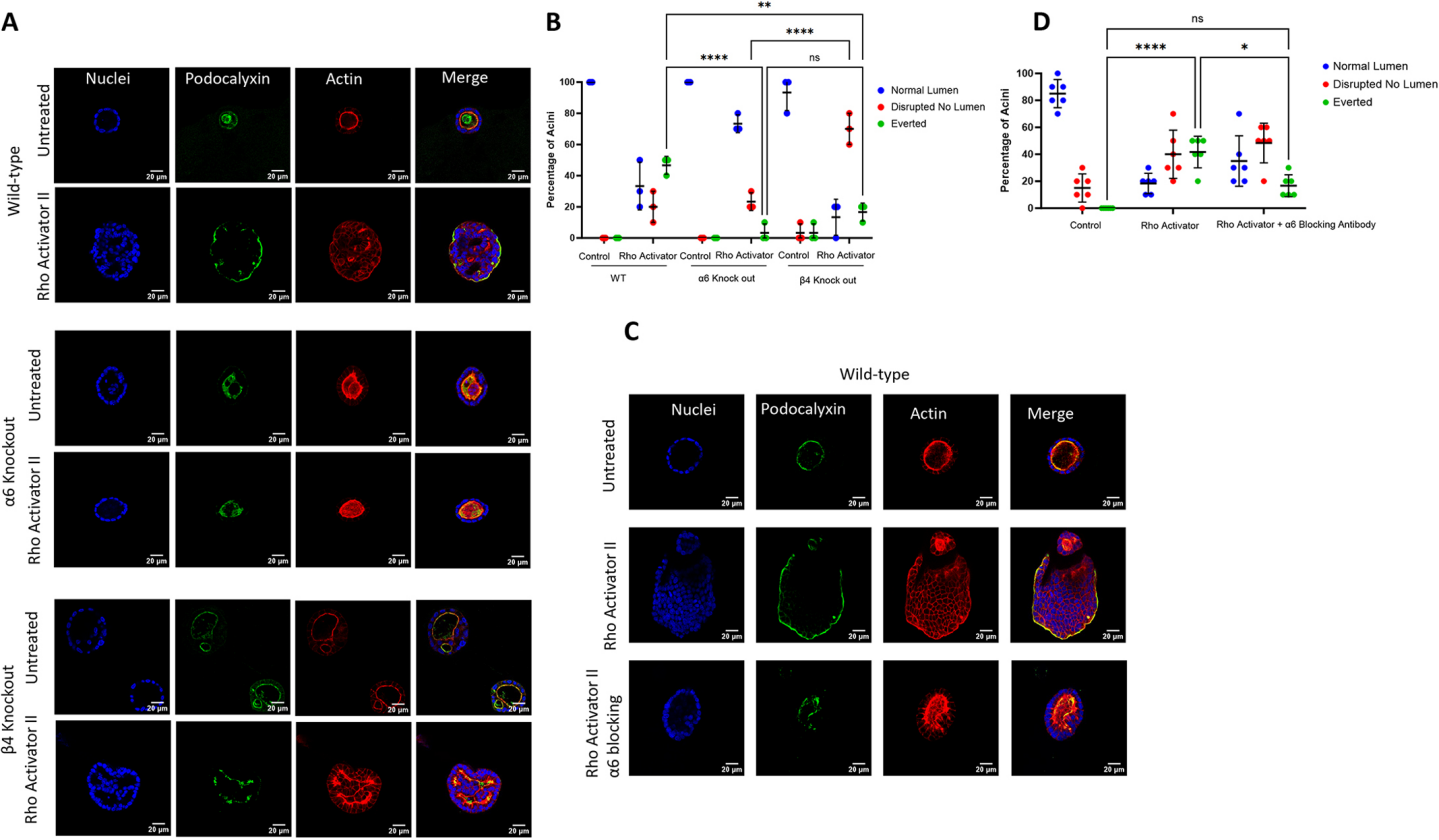
**Loss of α6 or β4 integrins significantly inhibits RhoA-mediated eversion.** (A) MDCK cysts were formed by seeding WT, α6 integrin-knockout, or β4 integrin-knockout MDCK cell lines in Matrigel. MDCK cysts were then treated with RhoA activator II for 48 h. Immunostaining was performed for podocalyxin and actin (phalloidin) to assess polarity. (B) Cysts were categorized as normal (hollow cell-free lumen and normal apical-in polarity), disrupted lumen (loss of hollow lumen but normal apical-in polarity) or everted (lumen collapse and apical markers on the outer membrane of the cyst). Each data point represents the mean (expressed as a percentage) of each condition from each replicate (minimum of 10 cysts analyzed per condition); three experiments were performed in total (error bars are overall mean±s.d.). The incidence of RhoA activator II-induced eversion was significantly less in both α6 and β4 knockout cysts. (C) MDCK cysts were left untreated, or treated with α6-blocking antibody, RhoA activator II or α6 blocking antibody with RhoA activator II, and then fixed and immunostained. (D) Quantification of normal, disrupted lumen and eversion for blocking antibody experiments. Each data point represents the mean percentage of each condition from each experimental replicate (minimum of 10 cysts analyzed per condition), six experiments were performed in total (error bars are overall mean±s.d.). *P<0.05; **P<0.01; ****P<0.0001; ns, not significant (two-way ANOVA with Tukey's multiple comparison test).

Our previous studies of RhoA-driven changes in apical-basal polarity have shown that eversion is a collective process that requires cell–cell adhesions (Narayanan et al., 2023). To test whether collective migration of cells is important for eversion, we examined whether specific integrins were important in this process, focusing on a potential role for laminin-binding α6β4 integrin (hereafter denoted α6β4). We screened a set of existing α6- and β4-knockout MDCK cell lines (Myllymäki et al., 2018; Zhang et al., 2017) for their ability to undergo RhoA-driven eversion. Apical polarity of MDCK spheroids was assessed using both actin and podocalyxin (gp135; also known as PODXL) immunostaining (Fig. 1A; Fig. S1). α6- and β4-knockout spheroids exhibited lumen collapse following Rho activator II treatment (Fig. 1A,B), indicating that lumen collapse is not dependent on these integrins. β4-knockout spheroids had more lumen collapse (disrupted lumen) when compared to α6-knockout spheroids. Interestingly, α6- and β4-knockout cell lines exhibited significantly reduced incidences of apical-out polarity following 48 h of Rho activator II treatment (Fig. 1A,B), indicating that these integrins are necessary for the establishment of apical-out polarity and completing the eversion process.

To further test the importance of α6 integrins for eversion, we used the GoH3 functional rat α6-blocking antibody (Lotz et al., 1990). Specificity of the α6-blocking antibody was assessed by incubating this antibody for 48 h with living MDCK cysts, followed by fixation and immunostaining with an anti-rat-IgG secondary antibody. The α6-blocking antibody localized to the basolateral regions of wild-type (WT) MDCK cysts (Fig. S1) confirming the ability of the blocking antibody to bind to MDCK cysts in Matrigel. Furthermore, no immunolabeling of the blocking antibody was detected in α6 KO cysts, confirming that the antibody is specific for α6 integrins. MDCK cysts treated with α6-blocking antibody at the time of Rho activator II treatment did not develop apical out polarity (Fig. 1C,D). We also confirmed that Rho activator II treatment did not impact the ability of the α6-blocking antibody to bind to MDCK cysts (Fig. S1). We repeated these experiments using Caco-2 cysts, a human epithelial cell line that also assembles into spheroids with apical-in polarity. First, we confirmed that Caco-2 cells, when treated with Rho activator II, developed apical-out polarity (Fig. S2). Next, we showed that treatment with α6-blocking antibody also inhibited apical-out polarity mediated by Rho activator II (Fig. S2).

**Fig. 2.**
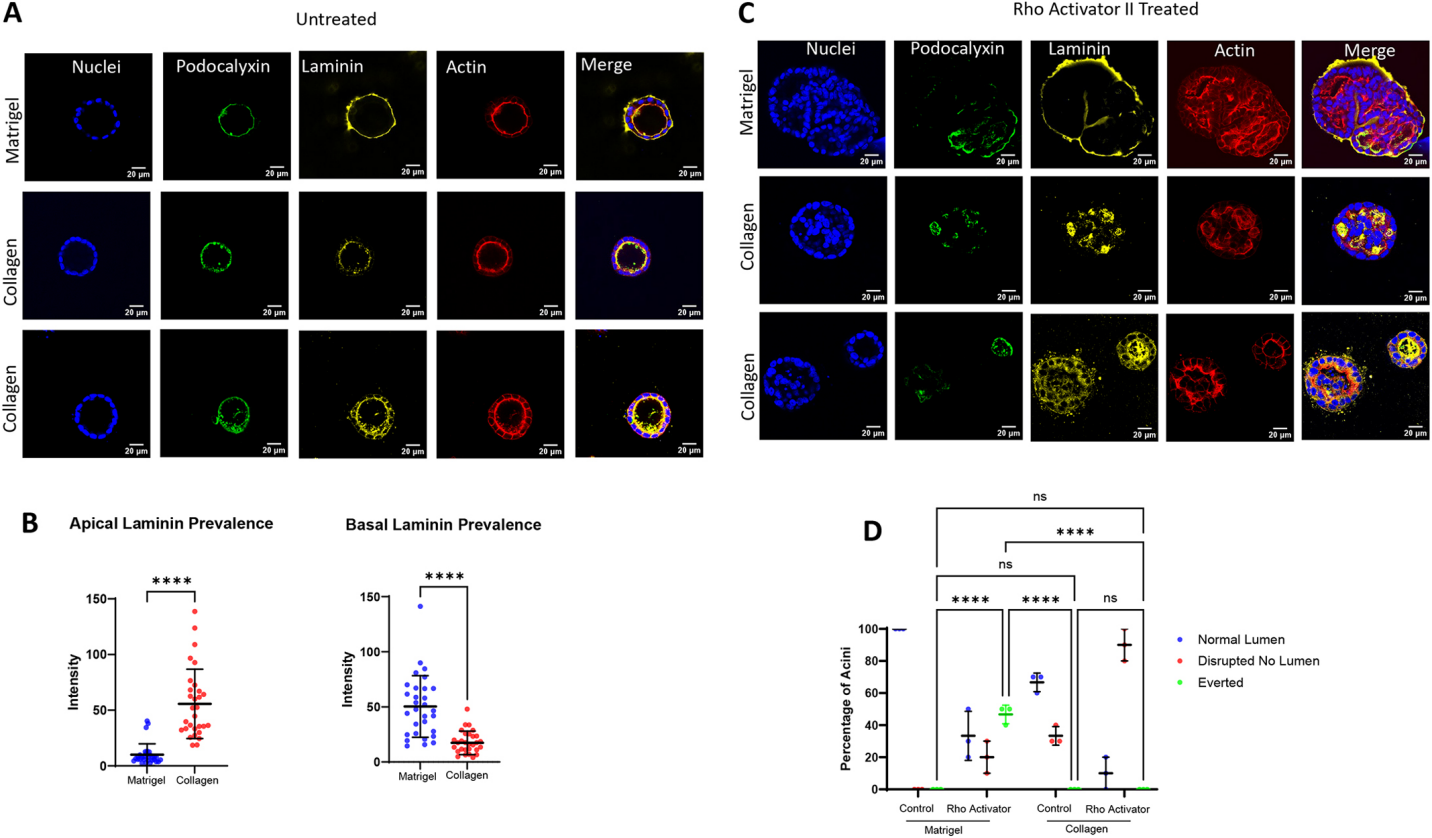
**Cells do not evert in collagen.** (A) MDCK cells were seeded into Matrigel or collagen and allowed to form cysts. Cysts were then fixed and immunostained for podocalyxin, laminin and actin (phalloidin). Less laminin basal laminin staining was observed for spheroids in collagen. (B) The relative intensity of apical and basal laminin immunostaining was quantified from multiple cysts (each data point represents one cyst). Error bars are mean±s.d. (C) MDCK cysts were treated with 48 h of RhoA activator II and then fixed and immunostained for podocalyxin, laminin, and actin (phalloidin). (D) Quantification of normal, disrupted lumen, and eversion for each condition. Each data point represents the mean average (expressed as a percentage) of each condition from each experimental replicate (minimum of 10 cysts analyzed per condition), three experiments were performed in total (error bars are overall mean±s.d.). ****P<0.0001; ns, not significant (two-way ANOVA with Tukey's multiple comparison test).

Because α6 and β4 integrins are important in the formation of laminin-binding hemidesmosomes, we sought to understand whether there would be differences for cells grown in Matrigel versus collagen gels. Matrigel contains laminin whereas the collagen used does not contain any additional ECM proteins, including laminin. We compared the formation of laminin ECM for MDCK cells grown in Matrigel versus collagen I ECM. MDCK spheroids formed a hollow lumen with correct apical-basal polarity in both Matrigel and collagen. When laminin ECM was assessed using an anti-laminin antibody, spheroids cultured in Matrigel had strong immunostaining for laminin at the basal (outer) surface (Fig. 2A,B). In contrast, spheroids cultured in collagen had a mixed phenotype in which the laminin network was either observed on the basal (outer) side of the spheroid or instead as assembled laminin at the apical surface (Fig. 2A,B). Regardless of the location of laminin, we observed much weaker immunostaining of laminin for cells cultured in collagen as compared to that seen in cells cultured in Matrigel (Fig. 2A). Prior reports have shown assembly of laminin on the basal surface for MDCK in collagen gels (O'Brien et al., 2001; Yu et al., 2005), although these prior studies did not directly compare laminin assembly for spheroids in collagen versus Matrigel. The reason for the inconsistent assembly of laminin at the basal (outer) surface of cysts in our collagen gels is not clear but might involve different sources of collagen. Nevertheless, the reduced basal laminin assembly in collagen ECM provided us with the opportunity to explore how this would affect eversion. We treated spheroids in Matrigel or collagen gels with Rho activator II (Fig. 2C). Although spheroids in collagen exhibited a disrupted lumen structure, we were not able to find any instances of apical-out polarity for spheroids in collagen treated with Rho activator II (Fig. 2C,D). We also tried to determine whether collagen-only ECM would suppress Rho-driven eversion for Caco2 spheroids; however, Caco2 cells did not properly develop into spheroids when cultured in pure collagen (data not shown). Overall, our results in Figs 1 and 2 show that α6β4-laminin interactions are necessary for establishment of apical-out polarity.

Given our data showing that α6- and β4-knockout cysts do not evert following Rho activator II treatment, we speculated that eversion could be a two-step process: (1) breaking of cell–cell adhesions, and (2) a collective migration of epithelial cells which requires α6β4–lamin interactions. To examine whether loss of α6 integrins reduces the mechanical forces in cysts (possibly limiting the ability for Rho activation to break cell–cell adhesions) we analyzed membrane tension analysis using the Flipper-TR membrane tension probe, in which higher fluorescence lifetime indicates higher membrane tension (Fig. S3). We observed that apical membrane tension was significantly higher than basal membrane tension across all conditions, which is consistent with a prior report showing increased tension at the apical surface in 2D MDCK monolayers (Roffay et al., 2024). Although untreated α6-knockout cysts exhibited significantly lower apical and basal membrane tension, treatment with Rho activator II restored membrane tension to WT levels, indicating that α6-knockout cysts do not have reduced membrane tension following Rho activation. Next, to directly assess whether breaking of cell–cell adhesions would trigger eversion in α6-knockout cysts, we performed laser ablation experiments. Previously, we have shown that laser ablation is sufficient to disrupt cell–cell adhesions in pressurized cysts and trigger eversion (Narayanan et al., 2023). In agreement with our prior published findings, we observed four out of five WT cysts undergo eversion following laser ablation (Fig. S4). However, no α6-knockout cysts everted following ablation (*n*=9). The differences in eversion between WT and α6-knockout cysts were significant based on the Fisher's exact test (*P*=0.005). These results further support our hypothesis that breaking of cell–cell adhesions alone is insufficient for eversion, but instead that α6β4 integrins are also necessary for eversion.

### Apical-out spheroids can return to apical-in polarity through apoptotic cavitation

**Fig. 3.**
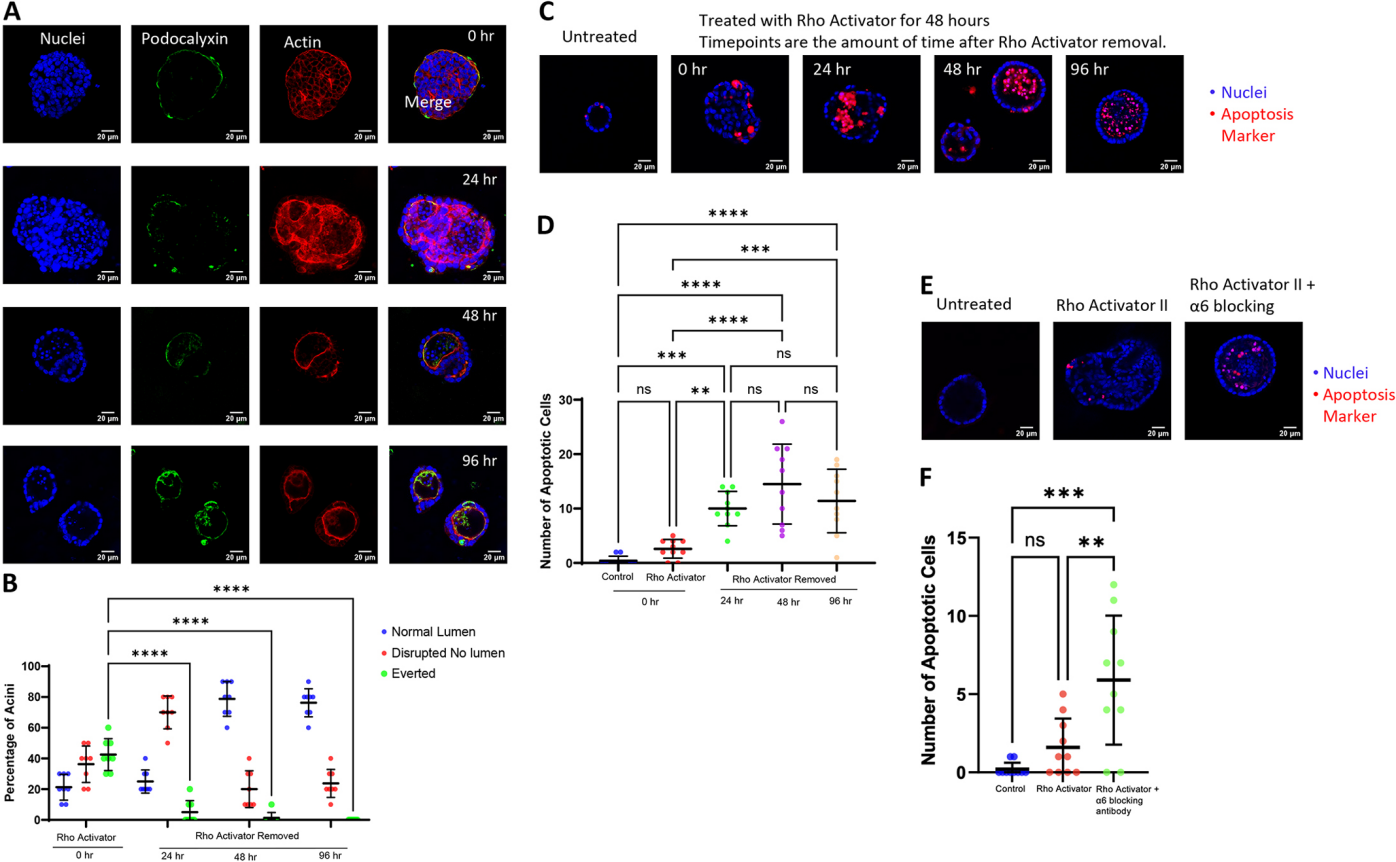
**Reversion of apical-out polarity to apical-in polarity occurs through apoptotic cavitation.** (A) MDCK cysts were grown in Matrigel for 7 days, and then treated with RhoA activator II for an addition 48 h. RhoA activator II was removed from culture through a change of medium. Cysts were fixed and immunostained at the indicated time points where time indicates the amount of time after RhoA activator removal. (B) Quantification of normal, disrupted lumen and eversion for each timepoint. Each data point represents the mean (expressed as a percentage) of each condition from each experimental replicate (minimum of 10 cysts analyzed per condition), eight experiments were performed in total across each condition (error bars are overall mean±s.d.). (C) Apoptosis was assessed using TUNEL assay at each timepoint. (D) Quantification of the number of apoptotic cells per cyst, each data point represents the mean of the number of apoptotic cells per cyst (total of 10 cysts per condition analyzed) (error bars are overall mean±s.d.). (E) MDCK cysts were left untreated, treated with RhoA activator II, or RhoA activator II and α6-blocking antibody and apoptosis was assessed with the TUNEL assay. (F) Quantification of the mean of apoptotic cells per cyst, each data point represents one cyst (total of 10 cysts per condition analyzed). Error bars are mean±s.d. **P<0.01; ***P<0.001; ****P<0.0001; ns, not significant (two-way ANOVA with Tukey's multiple comparison test).

We sought to understand whether eversion (changing from apical-in to apical-out polarity) is a reversible process. Spheroids were treated for 48 h with Rho activator II to induce eversion, and then the medium was replaced with fresh medium (without Rho activator II). After 24 h there was a statistical decrease in the number of spheroids with apical-out polarity, and a significant decrease in lumen disruption was observed at 48 h (Fig. 3A,B). These data indicate that eversion is a reversible process. Following removal of Rho activator II, we also observed altered nuclear morphologies for cells in the center of the spheroids (Fig. 3A) that were suggestive of apoptosis. To assess apoptosis, we used the TUNEL assay, which detects fragmented DNA in cells. Following removal of Rho activator II, we observed an increase in apoptotic cells (Fig. 3C). Notably, these apoptotic cells were only observed at the center of the spheroid, and no apoptosis was observed for cells in the outermost layer in contact with ECM (Fig. 3C). We also examined whether treatment with α6-blocking antibody was sufficient to restore a hollow lumen. Spheroids were first treated with RhoA activator II, followed by treatment with α6-blocking antibody (in the presence of RhoA activator II). Spheroids treated with α6-blocking antibody also exhibited increased apoptosis at the center of the spheroid (Fig. 3D). These data indicate that eversion is a reversible process, that hollow lumens are re-established by apoptotic cavitation and blocking α6 integrins is sufficient to stimulate apoptotic cavitation.

### Apical-out polarity is maintained by anchorage independence

**Fig. 4.**
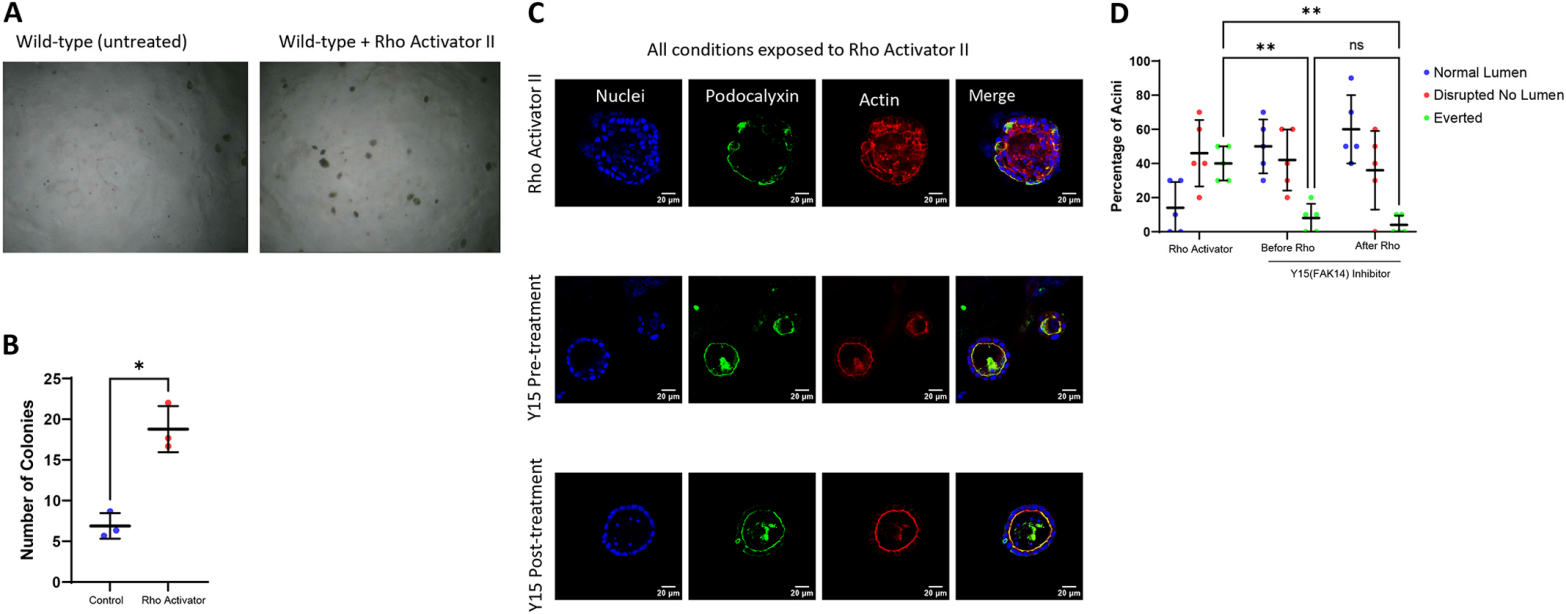
**Anchorage independence is required for apical-out polarity.** (A) MDCK cells were seeded into soft agar. Cells were left untreated or were treated with RhoA activator II. (B) Quantification of colonies formed in the agar assay. Each data point represents the mean (from a minimum of three field of view) from each experimental replicate (three replicate experiments were performed in total) (error bars are overall mean±s.d.). *P<0.05 (paired two-tailed *t*-test). (C) MDCK cysts were grown in Matrigel for 7 days and then treated with RhoA activator II for 72 h, pre-treated with FAK inhibitor Y15 24 h followed by Y15+Rho Activator II for an additional 48 h, or treated with Rho activator II for 24 h, followed by Y15+Rho activator II for an additional 48 h (post treatment). (D) Quantification of normal, disrupted lumen and eversion for each condition. Each data point represents the mean (expressed as a percentage) of each condition per replicate experiment (minimum of 10 cysts analyzed per condition per experiment, five replicate experiments performed in total; error bars are overall mean±s.d.). **P<0.01; ns, not significant (two-way ANOVA with Tukey's multiple comparison test).

As we had observed that transitions from apical-out to apical-in polarity occur through apoptosis, we hypothesized that apical-out spheroids might exist by cells developing anchorage independence (resistance to anoikis). To directly measure anchorage-independent cell growth, we used a soft agar colony-forming assay. Rho activator II-treated cells exhibited increased number and size of colonies in soft agar (Fig. 4A,B). Next, we treated spheroids with an inhibitor of focal adhesion kinase (FAK; also known as PTK2) as inhibition of FAK has been shown to restore anchorage dependence (Ward et al., 2012). Pre-treatment of spheroids with FAK inhibitor prevented infilling of the lumen with cells (Fig. 4C,D). Additionally, treatment of everted apical-out spheroids with FAK inhibitor also reduced the infilling of the lumen with cells (Fig. 4C,D). In both cases the FAK inhibitor (pre-treatment or post-treatment) appeared to drive formation of an apical-in actin network; however, podocalyxin staining exhibited an intermittent staining pattern (Fig. 4C,D). We also confirmed that the FAK inhibitor did not have a significant inhibitory effect on proliferation (Fig. S5), indicating that inhibition of proliferation is not the mechanism by which the FAK inhibitor prevents eversion. We conclude that FAK inhibitors can restore hollow lumens, possibly by suppressing anchorage independence, but can only partially restore apical-in polarity.

### Apical-out polarity is maintained by increased proliferation

**Fig. 5.**
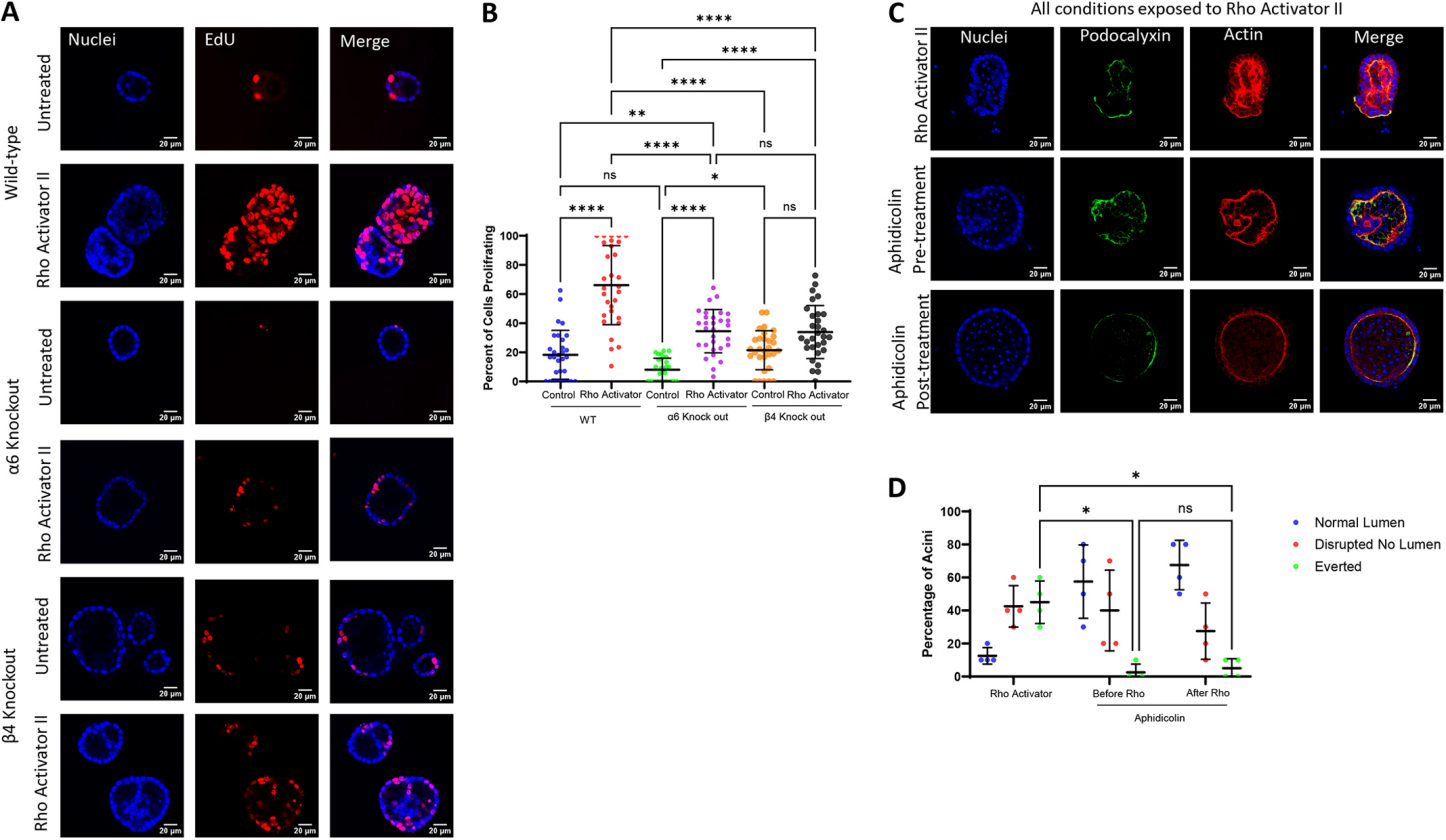
**Increased cell proliferation is required to maintain apical-out polarity.** (A) MDCK cells (WT, α6-integrin knockout, or β4 integrin-knockout) were grown in Matrigel for 7 days. Cells were left untreated or treated with RhoA activator II for an additional 48 h. Cells were incubated with EdU for the final 24 h of RhoA treatment. Cells were then fixed and EdU incorporation assed using anti-EdU antibodies. (B) Quantification of the percentage of cells with EdU incorporation. Each data point represents the mean (expressed as a percentage) of cells proliferating in a single cyst. Three separate experiments, with 10 cysts analyzed per experiment, were performed in total across each condition (error bars are overall mean±s.d.). (C) MDCK cysts were grown in Matrigel for 7 days and then treated with RhoA activator II for 72 h, pre-treated with DNA synthesis inhibitor aphidicolin for 24 h followed by aphidicolin+Rho activator II for an additional 48 h, or treated with Rho activator II for 24 h to induce eversion, followed by aphidicolin+Rho activator II for an additional 48 h (post treatment). (D) Quantification of normal, disrupted lumen and eversion for each condition. Each data point represents the mean average (expressed as a percentage) of each condition from each replicate (minimum of 10 cysts analyzed per condition, four replicate experiments in total; error bars are overall mean±s.d.). *P<0.05; **P<0.01; ****P<0.0001; ns, not significant (two-way ANOVA with Tukey's multiple comparison test).

Because apical-out spheroids appeared to contain higher number of cells, we hypothesized that apical-out spheroids might have a higher proliferation rate. Using an EdU proliferation assay, we observed that Rho activator II treatment significantly increased incorporation of EdU into cells, indicating that these spheroids had increased proliferation (Fig. 5A,B). To investigate whether RhoA-treatment itself (versus apical-out polarity) is the driver of increased proliferation we also performed the EdU assay in Rho activator II treated α6- and β4-knockout cells. α6- and β4-knockout cells had a significant increase in cell proliferation following Rho activator II treatment; however, this increase in proliferation was significantly lower as compared to WT cells (Fig. 5A,B). Thus, we conclude that transitions to apical-out polarity further enhance Rho-induced increases in proliferation. Next, we assessed whether inhibiting cell proliferation would affect apical-out polarity. Treatment of spheroids with aphidicolin before Rho Activator II treatment or after eversion resulted in spheroids with normal actin polarity and reduced cell infilling (Fig. 5C,D). Similar to FAK inhibition (Fig. 4C), aphidicolin also resulted in an irregular podocalyxin staining, suggesting that inhibition of polarity only partially restores apical-basal polarity.

## DISCUSSION

**Fig. 6.**
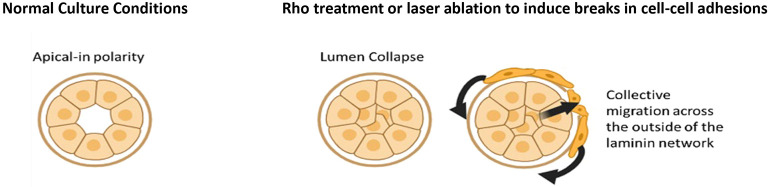
**Proposed mechanism for eversion.** Epithelial cells in Matrigel or collagen develop with apical-in polarity, forming a single hollow lumen. Local breaks in cell–cell adhesions can induce a rapid collapse of the lumen. This is then followed by a collective migration, using α6β4 interactions across the laminin network that everts the spheroid.

Our study provides new mechanistic insights into the mechanisms that drive eversion. In this study, we demonstrated that spheroids lacking α6 or β4 integrins, inhibition of α6 with a blocking antibody and ECM lacking laminin prevent spheroids from transitioning to apical-out polarity following treatment with Rho activator II (Figs 1 and 2). We also show that α6-knockout spheroids do not evert following laser ablation (Fig. S4). Thus, we conclude that although eversion might be initiated by breaks in cell–cell adhesions that lead to lumen collapse, that α6β4 integrins and laminin are required to transition to apical-out polarity through a potential collective migration of cells (mechanism summarized in Fig. 6). The collective nature of eversion is further supported by our prior studies showing that a blocking antibody for E-cadherin inhibited eversion (Narayanan et al., 2023). We also demonstrate that apical-out polarity is maintained through increases in cell proliferation and the loss of anchorage-dependence (Figs 4 and 5). Interestingly, spheroids with apical-out polarity can revert to apical-in polarity when RhoA activation is stopped (Fig. 3A), when cell proliferation is inhibited (Fig. 5C), when spheroids are treated with α6-blocking antibody (Fig. 3D), or when focal adhesion kinase activity is inhibited (Fig. 4C), showing that epithelial spheroids can readily transition between apical-in and apical-out polarity.

Our study presents the concept that inverted (apical-out) polarity is associated with more aggressive epithelial cell phenotypes, including increased proliferation and anchorage independence. It is an open question whether apical-out polarity itself promotes these cellular phenotypes. An additional question is whether our spheroid model of apical-out polarity has relevance to incidences of *in vivo* apical-out polarity, which includes subsets of carcinomas, including invasive micropapillary carcinoma (IMPC). IMPC is associated with increased metastases to local lymph nodes and lymphovascular invasion, with apical-out polarity most often observed at the invasion front (Guzińska-Ustymowicz, 2014; Nassar, 2004; Onuma and Inoue, 2022). The increased incidence of lymphatic invasion of cancers with inverted apical-basal polarity strongly suggests that clusters of cells with apical-out polarity are advantaged for invasion and metastatic spread of cancer. It is tempting to speculate that changes in apical-basal polarity orientation might affect the composition of signaling receptors on the outermost surface of the spheroid. If true, we would expect that apical-out spheroids would have different signaling in responses to soluble ligands when compared to spheroids with apical-in polarity.

Our results also show that apical-out polarity is sustained in part by increased cell proliferation (Fig. 5) and anchorage independence (Fig. 4). Restoration of anchorage-dependence (through FAK inhibition) or inhibition of proliferation was sufficient to promote clearance of cells from the lumen and a partial return to normal apical-in polarity. We therefore conclude that anchorage-dependent apoptotic clearance is an important process for maintaining a cell-free hollow lumen. It might therefore be notable that the phenotypes we identified associated with apical-out polarity (α6β4 integrins, anchorage independence, increased cell proliferation) have all been independently associated with cancer progression and invasion (Aoudjit and Vuori, 2012; Deng et al., 2021; Feitelson et al., 2015; Guo et al., 2006; Humphries et al., 2022; Weaver et al., 2002; Zahir et al., 2003). Future work is needed to determine whether changes in apical-basal polarity of carcinomas promote anchorage independence and increased proliferation.

An important result of our studies is that epithelial cells that have developed apical-out polarity (along with a cell-filled lumen) can readily revert to apical-in polarity (including returning to a hollow cell-free lumen) once the eversion-driving or apical-out-promoting stimuli are eliminated (Fig. 3). This demonstrates that apical-out polarity is not permanent, but instead that inverted spheres can return to normal apical-in polarity. It would be interesting to determine whether apical-out carcinomas can also return to apical-in polarity, and whether this conversion to apical-in polarity could suppress the invasiveness typically observed in apical-out carcinomas (Nassar, 2004; Zajac et al., 2018).

Our results demonstrate that the ‘reversion’ from apical-out to apical-in polarity occurs through apoptotic clearance of cells located in the center of the spheroid (Fig. 3C). We note that this mechanism appears similar, if not identical, to apoptotic cavitation that has been observed during developmental processes that drive formation of epithelial tubular structures (Lubarsky and Krasnow, 2003). Additionally, Mostov and colleagues have shown that apoptotic cavitation is an additional mechanism by which lumen formation occurs in *in vitro* spheroids (Martín-Belmonte et al., 2008; O'Brien et al., 2002).

In summary our work further elucidates how apical-out polarity is established and maintained in epithelial spheroids, highlighting the importance of α6β4 integrins and laminin in polarity transitions, as well as the importance of anchorage independence and proliferation in maintaining the apical-out polarity state.

## MATERIALS AND METHODS

### Cell lines and culture

MDCK II cells, originally a gift from Rob Tombes (Virginia Commonwealth University, Richmond, USA), and Caco-2 cells (HTB-37, ATCC) were used in these studies. To examine the role of specific integrins in the eversion process we utilized α6- and β4 integrin-knockout MDCK cells that were previously established from the MDCK-II Heidelberg strain (Myllymäki et al., 2018; Zhang et al., 2017). In experiments using integrin-knockout cells, they were exclusively compared to the parental MDCK-II Heidelberg strain. All cell lines were cultured in DMEM with 4.5 g/l glucose (Thermo Fisher Scientific), supplemented with 10% (v/v) fetal bovine serum (FBS, Thermo Fisher Scientific) and 1% (v/v) penicillin-streptomycin mix (pen-strep, Thermo Fisher Scientific). Cell lines were confirmed to be free of mycoplasma contamination using the Mycoplasma PCR detection kit (catalog #G238, Applied Biological Materials Inc.).

### EdU incorporation assay

For proliferation, the Click-iT™ Plus EdU Cell Proliferation Kit for Imaging, Alexa Fluor 488 (Thermo Fisher Scientific, catalog #C10637) was used. EdU was added to the cells 24 h prior to fixation. The next day the cells were fixed and stained as per the manufacturer's instructions.

### Inhibition experiments

Aphidicolin was used at a final concentration of 2 μg/ml (Sigma-Aldrich, catalog #A4487) to inhibit DNA synthesis. Focal adhesion kinase (FAK) inhibitor Y15 (also known as FAK inhibitor 14) was used at a final concentration of 2.5 μM (Medchem express, catalog HY-12444). In our pre-treatment experiments, the aphidicolin and Y15 were used for 24 h and then maintained for an additional 48 h with Rho activator treatment. In post-treatment experiments each inhibitor was added at 24 h post-Rho activator treatment for 48 h (with the Rho activator).

### Formation and culture of MDCK spheroids in Matrigel

For formation and culture of 3D spheroids in Matrigel, cells were trypsinized from tissue culture plates and 5000 cells were suspended in 400 μl of growth medium supplemented with 2% (v/v) Matrigel. The cell suspension was seeded onto an eight-well Nunc Lab-Tek II chambered coverglass or coverslip, pre-coated with 40 μl of phenol-free growth-factor-reduced (GFR) Matrigel (Corning) as previously described (Zhang et al., 2019). The pre-coated Matrigel was allowed to solidify by incubation for at least 30 min at 37°C before seeding the cells. The growth medium was changed every 3 to 5 days and the cells were allowed to form acini for 7–12 days. To induce eversion spheroids were treated with Rho activator II (Cytoskeleton) at a final concentration of 2–4 μg/ml for 48 h. In some batches of Matrigel lower concentrations (1–2 μg/ml) of Rho activator II did not induce eversion. Because of inconsistencies with eversion with different lot numbers of Matrigel, the Rho II activator concentration was chosen such that eversion consistently occurred in untreated WT cells without any evidence of loss of cell–cell cohesion [loss of cell–cell adhesion was previously observed at doses of 5 μg/ml or 10 μg/ml Rho activator II (Narayanan et al., 2023)].

### Formation and culture of MDCK spheroids in collagen

For formation and culture of 3D spheroids in collagen, cells were trypsinized from tissue culture plates and suspended in a collagen type 1 solution (PureCol catalog #5005, Advanced BioMatrix) and seeded onto eight-well Nunc Lab-Tek II chambered coverglass as previously described (Elia and Lippincott-Schwartz, 2009). Briefly, collagen solution was prepared by mixing 50 μl GlutaMax (200 mM), 625 μl NaHCO_3_ (2.35 mg/ml), 625 μl MEM 10×, 125 μl HEPES (1 M, pH 7.6), and 4.13 ml Collagen 1 (2 mg/ml) to a final volume of 6.255 ml at neutral pH. Eight-well chambers were coated with the collagen solution with 40 μl and incubated for polymerization in 37°C oven for 30 min. Cells were diluted into the collagen solution at 10,000 cells/ml, ensuring the cell volume did not exceed 10% of the total solution. 40 μl of the cell–collagen mixture was applied to pre-coated plates. The growth medium was changed every 3 to 5 days and the cells were allowed to form acini for 7–12 days. To determine whether spheroids cultured in collagen underwent eversion, spheroids were treated with Rho activator II (Cytoskeleton) at a final concentration of 3.5 μg/ml for 48 h.

### Formation and culture of Caco-2 spheroids

Caco-2 spheroids were formed similarly to MDCK spheroids, except that a mixture Matrigel and collagen was used (collagen I, final concentration of 1 mg/ml; Matrigel, final concentration of 40% or more) (Jaffe et al., 2008). On day 6 they were treated with cholera toxin (0.1 µg/ml, Sigma) to enhance lumen formation (Jaffe et al., 2008).

### α6-blocking experiments

WT cells were cultured in Matrigel within eight-well chambers. After 7–10 days of culture, the resulting acini were exposed to different experimental conditions. The control wells remained untreated, while others were treated with Rho activator II, or Rho activator II combined with the α6 functional blocking antibody (clone GoH3, BioLegend) at a concentration of 10 μg/ml. Following 48 h of treatment, the cells were fixed using 2% paraformaldehyde (PFA).

### Immunofluorescence

For immunofluorescence staining experiments, cells were fixed in 2% paraformaldehyde, followed by 0.1% Triton X-100 permeabilization. Primary antibodies used were against: podocalyxin/gp135 (1:250, clone 3F2:D8, Sigma), α6 integrin (1:300, clone GoH3, BioLegend), β4 integrin (1:300, clone M126, Abcam), laminin (1:250, catalog L9393, Sigma Aldrich), and phospho-ezrin/radixin/moesin (1:250, catalog 3726S, Cell Signaling). Anti-mouse-IgG secondaries (1:500) were used to label Gp135 and β4 integrin (Thermo Fisher Scientific, catalog A21202, A10037 and A31571), which allowed us to stain with Alexa Fluor 488, 568 and 647; anti-rabbit-IgG secondaries (1:500) were used to label ezrin, laminin and α6 integrin (Thermo Fisher Scientific, catalog A21206, A10042 and A31573, for Alexa Fluor 488, 568 and 647). Actin was labeled using fluorescently tagged phalloidin (Cytoskeleton). Nuclei were labeled with Hoechst from Thermo Fisher Scientific (catalog H3570). Cells were mounded with ProLong Gold Antifade Mountant (Thermo Fisher Scientific, catalog P36930) and imaged on a Stellaris 8 confocal microscope (Leica) with a 40× objective.

### TUNEL apoptosis assay

WT MDCK cells were seeded into Matrigel and cultured in eight-well Nunc Lab-Tek II chambered cover glasses for 7-12 days. Acini formation was observed within 7–10 days. Subsequently, the acini were treated with Rho Activator II for 48 h and fixed at various time points following the removal of Rho Activator II, specifically at 0, 24, 48 and 96 h. Cells were fixed using paraformaldehyde (PFA) and apoptosis was assessed using the Click-iT TUNEL Assay Kit (C10619, Thermo Fisher Scientific) per manufacturer instructions.

To evaluate the apoptotic effects of the α6 blocking antibody, WT cells were subjected to the following experimental conditions: a control group without Rho Activator II, a group treated with Rho Activator II, and a group treated with Rho Activator II in combination with the α6 blocking antibody. Cells were fixed using paraformaldehyde (PFA) and apoptosis was assessed using the Click-iT TUNEL Assay Kit (C10619, Thermo Fisher Scientific).

### Agar assay

To assess anchorage independence a standard agar assay was used (DeWane et al., 2023). WT cells were seeded in T25 flasks 2 days before the assay. One group remained untreated as the control, while the experimental group was pre-treated with Rho activator II at a concentration of 2 μg/μl. The day prior to the experiment, six wells were coated with a 1% F127 solution in PBS for 1 h to prevent cell attachment to the surface. Agarose (Thermo Fisher Scientific, catalog BP-165-25) solutions at concentrations of 4% and 1.75% were prepared in ultrapure water and medium containing 12% FBS was also prepared. To create the bottom layer, the 4% agarose solution was mixed with medium in a 1:8 ratio, and 2 ml of this mixture was used to coat each well. The plates were allowed to set for 1 h in the biosafety hood. Cells were detached from T25 flasks by trypsin, counted, and ∼15,000 cells were added per well. For the top layer, 8 ml of cell-suspended medium was combined with 2 ml of 1.75% agarose solution. A total of 2 ml of this mixture was applied on top of the bottom layer in each well and allowed to solidify for an additional 3 h. The following day, 1 ml of medium was added to each well every week, with the experimental group receiving medium supplemented with Rho activator II at a final concentration of 2 μg/μl (Rho activator II treatment was maintained during the 4–5 weeks of the agar assay). After 4–5 weeks colonies were visible, and images were taken with an optical microscope.

### Laser ablation experiments

Samples were pre-incubated with SPY555-FastAct (Cytoskeleton, Inc., cat. #CY-SC205; 1:1000 dilution) for 2 h prior to imaging. Actin was used to visualize the apical membrane and to assess whether eversion occurred.

Laser ablation was performed using an Olympus FVMPE-RS multiphoton laser scanning microscope equipped with a 25×/1.05 NA water-immersion objective (XLPLN25XWMP2) and tunable Spectra-Physics InSight X3 femtosecond laser (80 MHz, >2 W). The laser was operated at 920 nm and 100% power at the laser head, with a pixel dwell time of 10, 20 or 40 μs, corresponding to frame times of 50.8, 53.9 or 77.9 ms respectively. Ablation was achieved by stimulating an elliptical ROI over the target area 4–6 consecutive times.

Live imaging post-ablation was performed on an Olympus FV3000 confocal laser scanning microscope using either a 10×/0.4 NA (UPLXAPO10X) or 20×/0.75 NA (UPLXAPO20X) air objective for 8 h, with 10-min interval between scans. The samples were maintained in a Tokai-Hit on-stage incubator at 37°C and 5% CO_2_.

### Flipper-TR membrane tension measurements

The Flipper-TR kit (cat. #CY-SC020, Cytoskeleton) was utilized for membrane tension analysis. The membrane tension probe was diluted to 1 μM in cell culture medium and added to acini overnight. The medium was swapped to a live-cell imaging solution prior to imaging. The lifetime of the membrane tension sensor was collected through fluorescence-lifetime imaging microscopy (FLIM,) and then ROIs were drawn for the apical and basal surfaces of the acini for collection. Data was aggregated across three biological replicates with at least 20 acini measured for each condition in each replicate.

### Statistics

For each spheroid eversion experiment, 10 spheroids per condition were scored as normal, lumen collapse, or eversion through the assessment of apical markers. Repeat experiments (on different days) were performed. Statistical differences were assessed using *P<0.05, **P<0.01, ***P<0.001, ****P<0.0001 by two-way ANOVA with Tukey's multiple comparison test. For the agar assay, a paired two-tailed *t*-test was performed.

## Supplementary Material



10.1242/joces.264323_sup1 Supplementary information
