# Supplementary material for: Apical-out polarity in epithelial spheroids requires α6β4 integrins, cell proliferation and anchorage independence
Source: J Cell Sci. 2026 Jul 13;139(13):jcs264323. doi: 10.1242/jcs.264323 (PMC13405224; doi:10.1242/jcs.264323)
Supplement: Supplementary information [file joces-139-264323-s1.pdf]

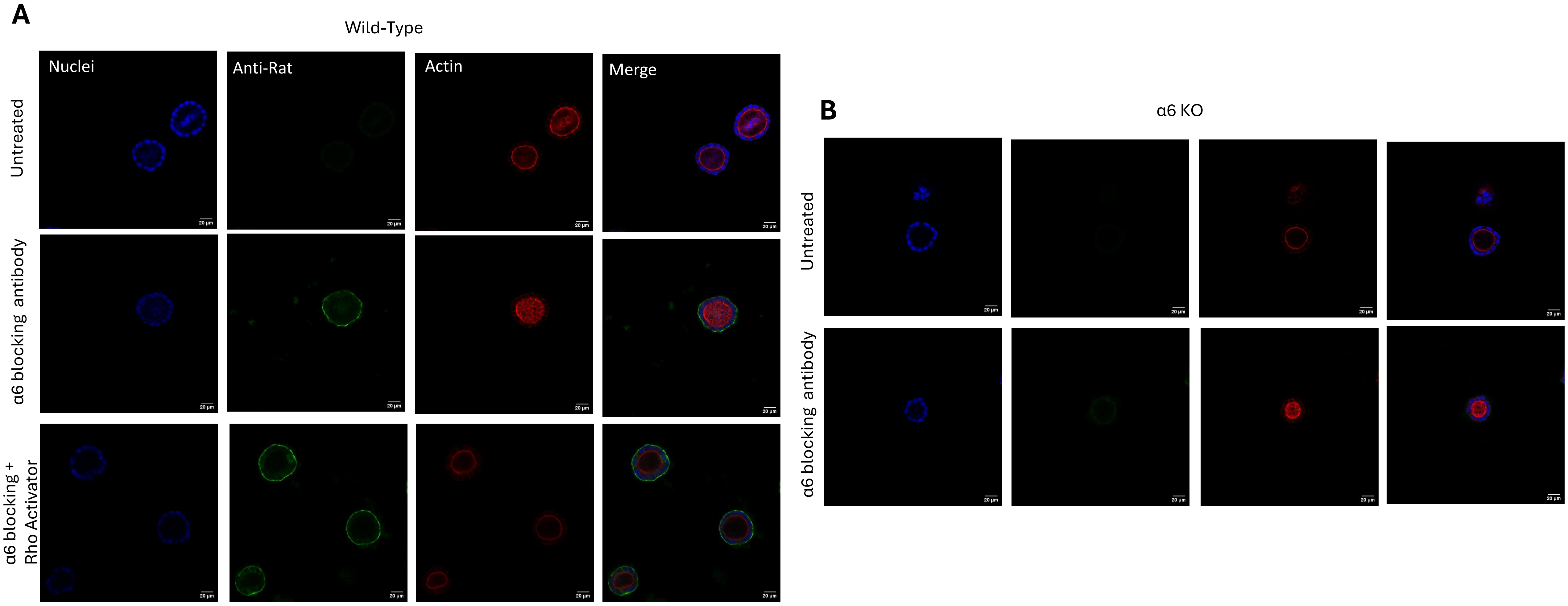

Fig. S1. Assessing α6 blocking antibody specifically in MDCK cysts. A) MDCK cysts were left untreated, or treated with rat α6 blocking antibody, or treated with rat α6 blocking antibody and RhoA Activator II. Binding of blocking antibody to cyst was assessed with immunostaining of rat blocking antibody with anti-rat secondary antibody. **B)** α6 KO cysts were left untreated or with α6 blocking antibody. Immunostaining shows no binding of blocking antibody for α6 KO cells.

Journal of Cell Science • Supplementary information

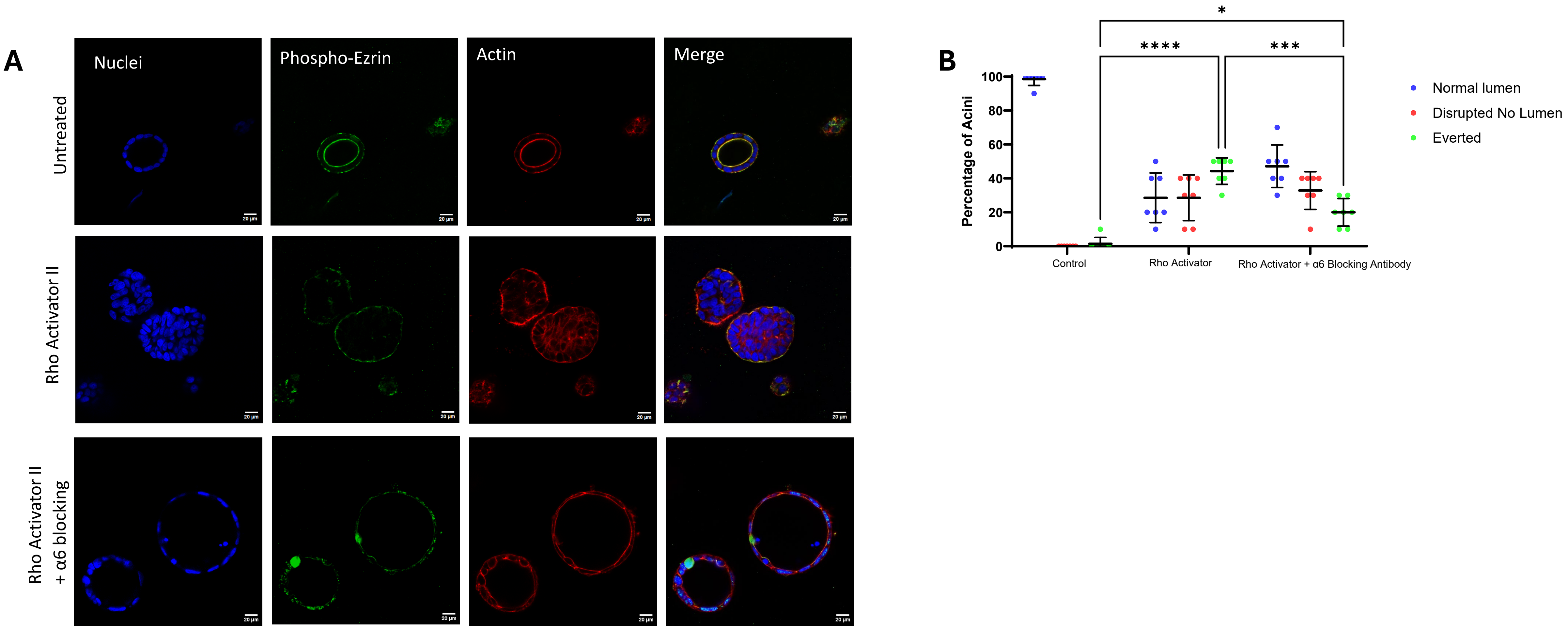

Fig. S2. Eversion of Caco-2 cells requires α6 integrins. A) Caco-2 cells were seeded in a mixture of collagen and Matrigel. Mature cysts were left untreated, or treated with α6 blocking antibody, RhoA activator II, or α6 blocking antibody with RhoA activator II. Cysts were fixed and immunostained for phospho-Ezrin and actin (phalloidin). **B)** Quantification of normal, disrupted lumen, and eversion for blocking antibody experiments. Each data point represents the average percentage of each condition per experiment (minimum of 10 cysts analyzed per condition), 6 experiments were performed in total across each condition.

Journal of Cell Science • Supplementary information

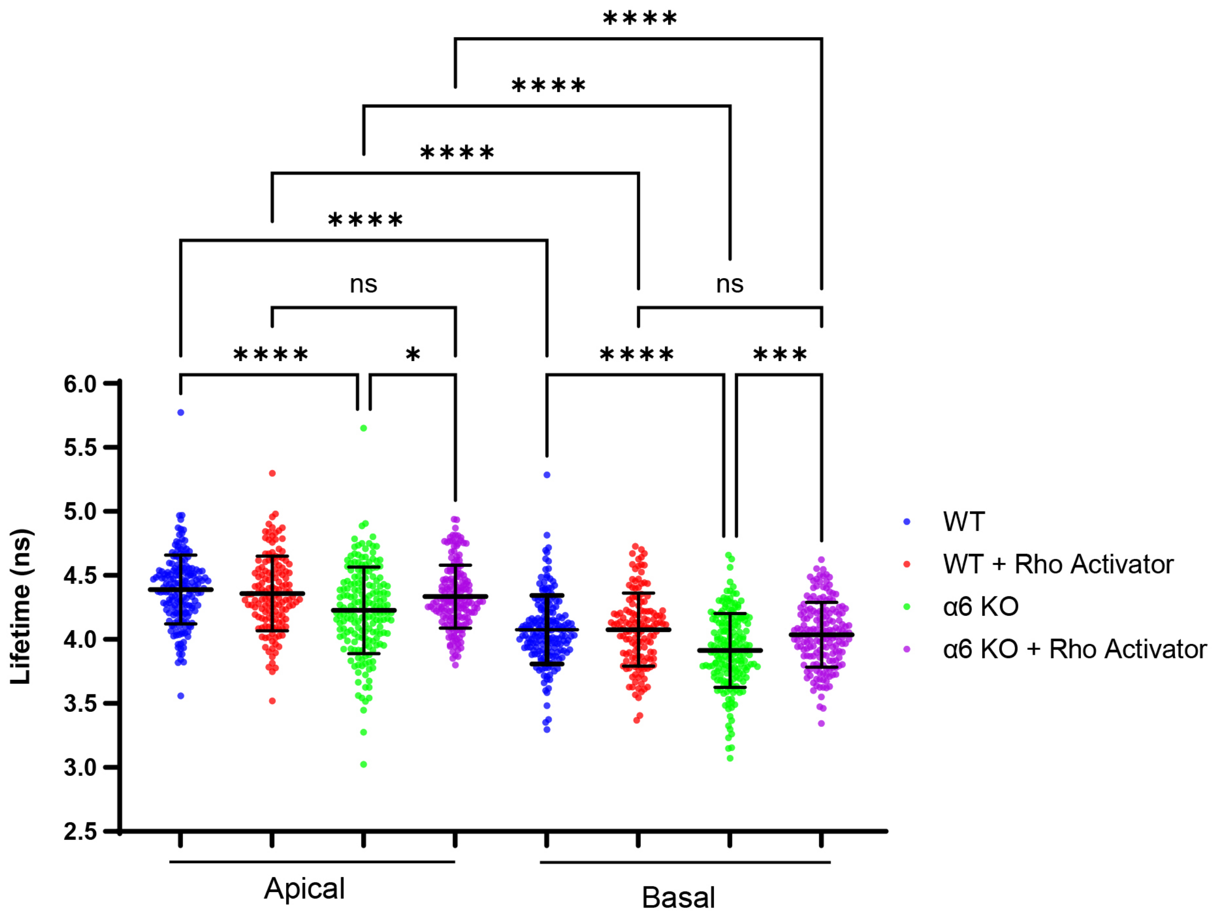

Fig. S3. Flipper-TR membrane tension measurements. Flipper-TR Kit (Cat. #CY-SC020, Cytoskeleton Inc) was utilized for membrane tension analysis of wild-type and α6 knockout MDCK cysts. The lifetime of the membrane tension sensor was collected through FLIM, and then ROI were drawn for the apical and basal surfaces of the acini for collection. Data was aggregated across 3 biological replicates with at least 20 acini measured for each condition in each replicate.

Journal of Cell Science • Supplementary information

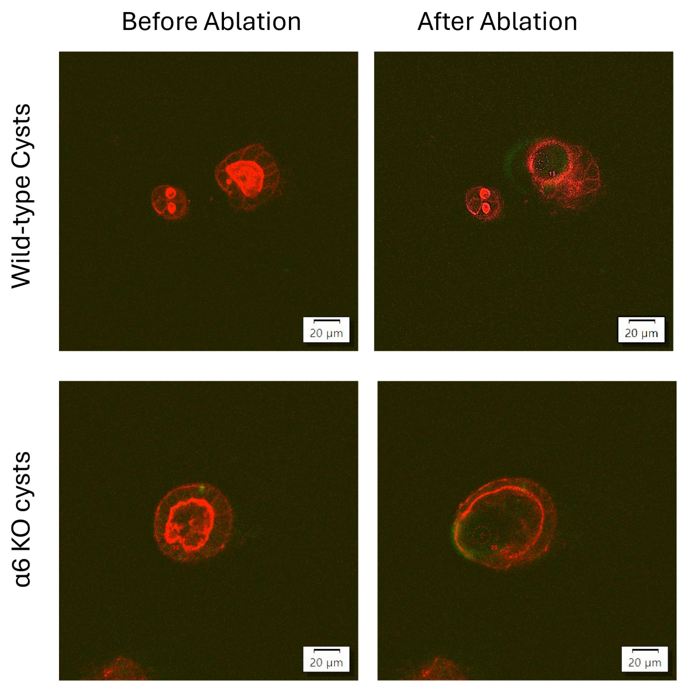

	Everted	Not Everted
Wild-type	4	1
A6 KO	0	9

p=0.0050, Fisher's Exact Test

Fig. S4. Assessment of eversion following laser ablation. MDCK cysts, labeled with SPY555-FastAct (red) were assessed before and after laser ablation. The site of laser ablation is shown with the dotted circle. Eversion was assessed as the presence of actin on the outer surface, as well as rearrangement of cells. In the case of α6 knockout cells, there were no changes in the localization of actin to the apical surface following laser ablation, whereas wild-type cells had 4 out of 5 cysts exhibit eversion.

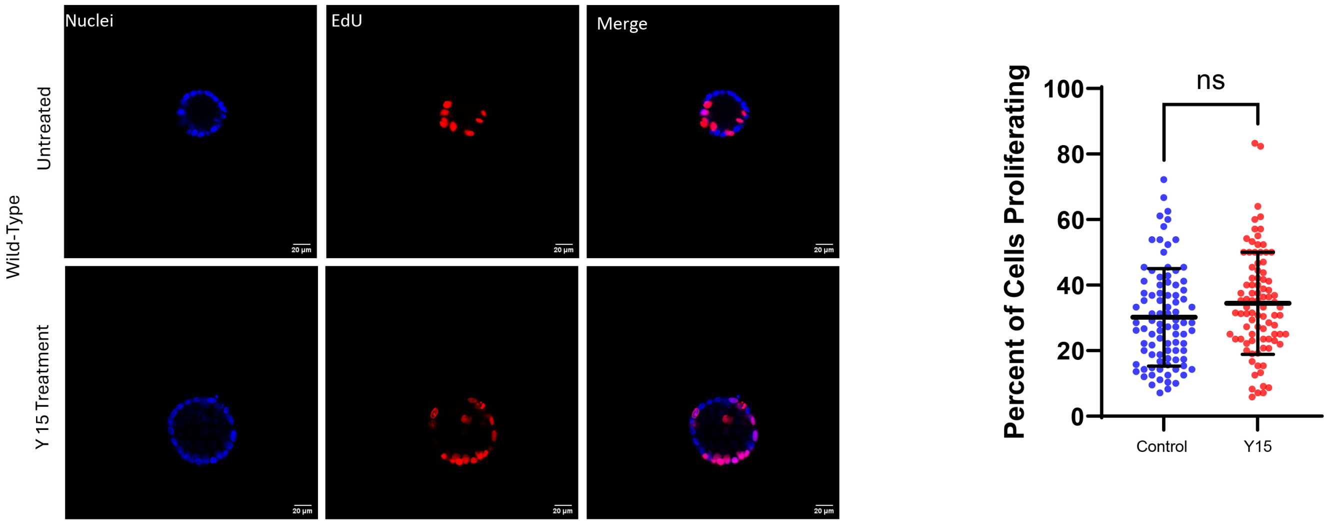

Fig. S5. Inhibition of FAK does not affect proliferation A) MDCK cysts were left untreated or with Y15 for 72 hours. EdU was added at 48 hours. Cells were fixed and EdU incorporation assessed using anti-EdU antibodies **B)** Quantification of the percent of cells with EdU. Each data point represents the average percentage of cells proliferating in a single cysts. Three separate experiments, with 10 cysts analyzed per experiment, were performed in total across each condition.
