# Supplementary material for: Apical-out polarity in epithelial spheroids requires α6β4 integrins, cell proliferation and anchorage independence
Source: J Cell Sci. 2026 Jul 13;139(13):jcs264323. doi: 10.1242/jcs.264323 (PMC13405224; doi:10.1242/jcs.264323)
Supplement: Supplementary file 1 [file joces-139-264323_review_history.pdf]

Apical-out polarity in epithelial spheroids requires α6β4 integrins, cell proliferation and anchorage independence

Niharika Patel, Jenna Pirrello, Skylar Lynch, Hailee Patel, Bradley J. Sweeney, Shivam Priya, Tanmay P. Lele, Aki Manninen and Daniel E. Conway
DOI: 10.1242/jcs.264323

Editor: Andrew Ewald

Review timeline

Original submission:	24 July 2025
Editorial decision:	3 September 2025
First revision received:	8 April 2026
Editorial decision:	18 May 2026
Second revision received:	28 May 2026
Accepted:	29 May 2026

Original submission

First decision letter

MS ID#: jcs.264323

MS TITLE: Epithelial eversion requires α6β4 integrins, cell proliferation, and anchorage-independence

AUTHORS: Niharika Patel; Jenna Pirrello; Shivam Priya; Aki Manninen; Daniel Conway

ARTICLE TYPE: Research Article

Dear Dr Conway,

We have now reached a decision on the above manuscript.

To see the reviewers' reports and a copy of this decision letter, please go to: View Reviewer Comments

As you will see, the reviewers are familiar with your past work and find the present study to be a useful addition to the field. However, they also raise a number of substantial criticisms that prevent me from accepting the paper at this stage. There are recurrent themes across both reviewers in terms of describing conclusions accurately and integrating the presented data into a more coherent narrative. There are also cumulatively significant specific points on mulitiple aspects of experimental design and analysis. Both reviewers are of the opinion that the RNA-seq, as presented, does not make a strong addition. I will leave it to your discretion whether it is better to integrate these data more fully or cut them. The reviewers suggest that a revised version might prove acceptable, if you can address their concerns. If you think that you can deal satisfactorily with the criticisms on revision, I would be pleased to see a revised manuscript. We would then return it to the reviewers.

Reviewer 1

© 2026. Published by The Company of Biologists under the terms of the Creative Commons Attribution License (https://creativecommons.org/licenses/by/4.0/).

This study is a follow up to a detailed study from 2023 in Biophys J by the same group that showed Rho activation triggers eversion of MDCK cell acini to an "apical-out" organization. Notably, they also showed that simple mechanical disruption of the acini by laser ablation was sufficient to trigger eversion, suggesting that Rho activation is not necessary but promotes eversion by creating local breaches in the junctions between the MDCK cells, through increased actomyosin contractility. These data make it a little unclear why they focus on integrin subunits and do not test if knocking out alpha6 integrin reduces these intercellular breaches. They should test the effect of laser ablation on the alpha6 KO cell acini.
Overall, the study fills in several details about the phenomenon of acinus eversion to an apical-out configuration, although many of the experiments seem to be unrelated to one another and mechanism underlying the requirement for alpha6 integrin is lacking.
Page 8 - it is unclear why the authors think an antibody (alpha blocking Ab) could penetrate through the tight junctions of the everted MDCK acini to affect the interior cells.
The authors note that FAK inhibition reduces infilling of lumens - could this be simply because of reduced cell proliferation? (since they observed reduced infilling when they treated with aphidocolin to inhibit proliferation). They should test this possibility, because if this is the case it is not interesting.
There are problems with the RNAseq - the authors ascribe changes in gene expression to eversion but it seems more likely they are caused principally by Rho activation - they have no good control for this and they do not test any of the potential hits. They try using alpha6 knockout cells as a control, but this knockout will have multiple other effects. In addition, their previous paper suggests that Rho activation is not the primary cause of eversion, but works because it damages intercellular junctions.
The authors also do nothing with these RNAseq data and it is unclear why they were included. They are not even mentioned in the Discussion. For these reasons I think that the RNAseq data should be removed from the manuscript.
Much of the Discussion is related to cancer and reversed polarity, but the treatment of MDCK cells in culture with a potent bacterial toxin to activate RhoA is decidedly unphysiological and the connection to cancer is tenuous at best. Much of this discussion could be removed.
Discussion - the authors state that "apical polarity is a marker for changes in epithelial cell phenotypes" - but I do not understand what this means. Isn't apical polarization itself a change in phenotype?

Minor and typographical issues:
The authors should add in the figure legends the meaning of the data points (biological replicates or technical replicates?) and the meaning of the ***s to signify significance (the statement "using *p<0.05 as per ANOVA" is insufficient).
page 5, line "Tunnel" 38 should be TUNEL.
page 7, line30 "MDCK cells with ("the "with" should be deleted.
page 7, line 40 "does not any" - need to add "contain"???
Page 8, line 17 "reversion" should be "eversion". Also on line 28.
Page 10, line 31, "feature epithelial cancers" should be "feature of epithelial cancers".

<u>Reviewer 2</u>

SUMMARY OF THE ADVANCE MADE IN THIS PAPER AND ITS POTENTIAL SIGNIFICANCE TO THE FIELD

The manuscript by Patel and colleagues examines the requirement for a6 and b4-integrins in the process of Rho activator-induced eversion of epithelial cysts in 3-dimensional culture. This builds on previous work from the authors and other laboratories understanding how epithelial cells can have alternate configurations of apical-basal polarity orientation.

The manuscript identifies a requirement for a6 and b4-integrins in the process of eversion upon RhoA activator treatment. Notably, the authors also indicate that eversion is accompanied with increased proliferation. This can be largely reverted by aphidicolin treatment to attenuate proliferation or treatment with a FAK inhibitor, providing some molecular insights into the process.

The work is largely descriptive and phenomenological. It contains little mechanistic insight into how these insights between Rho activation, integrin requirement, FAK signalling or proliferation increases are mechanistically linked. It compares Matrigel and Collagen, but these experiments

© 2026. Published by The Company of Biologists under the terms of the Creative Commons Attribution License (https://creativecommons.org/licenses/by/4.0/).

provide no mechanistic advance other than these two matrices being different. Despite these major shortcomings, I think a short, concise and not overclaiming manuscript could be of benefit for the field, if some items are removed from the manuscript, and quantitation provided. I detail these below.

SUGGESTIONS TO AUTHORS

Major comments [Please request additional experiments only if they are essential for supporting the conclusions; authors should be encouraged to highlight any claims that are preliminary or speculative, or to discuss any pitfalls or alternative interpretations in a 'Limitations' section]

-It would be very helpful at the start of the manuscript to help orient the reader by including a cartoon schema of the eversion process from your and others' previous work.

-You describe that "*a6 and β4* exhibited lumen collapse following Rho Activator II treatment (Figure 1A, 1B), indicating that lumen collapse is not dependent on these integrins. The quantitation in Figure 1B for b4-integrin KO displays an increase in disrupted lumen category, but whether this is significant is unclear. However, the image in Figure 1A for s6 KO does not seem to show disrupted lumen formation, nor does the quantitation in Figure 1B. Rather, it seems to show resistance to Rho Activator II effects. Therefore, this data is not described correctly. Therefore a6 and b4 appears to have distinct roles.
-Figure S1 lacks quantitation of phenotypes. All conclusions relating to effects of integrin KO in this figure require statistical support. Since these integrins are not studied further, in lieu of quantitation of these phenotypes, the authors could simply remove Figure S1 and the corresponding main text.
-Figure S2 lacks quantitation. Since the authors conclude "Î±6 blocking antibody had minimal effects on apical-basal polarity and did not disrupt single lumen morphology (Supplemental Figure 2)" the authors need to provide quantitation.
-Figure 1D lacks an antibody isotype control for anti-a6-integrin blocking antibody treatment. Moreover, the specificity of this antibody is not provided, such as lack of effect in a6 KO cells.
-In Figure 2A, you state "spheroids cultured in collagen had a mixed phenotype in which the laminin network was either observed on the basal (outer) side of the spheroid or instead as assembled laminin at the apical surface (Figure 2A)." Please provide quantitation.
I-n Figure 3C, D. Statements are made about the location and level of apoptosis. No quantitation is provided for assessment.
-In Figure 4B, please indicate if this represents 3 technical replicates of the same experiment, or 3 independent experiments.
I-n Figure 5B, please indicate if this represents multiple cysts from same experiment or from independent experiments. If independent, then it is appropriate to analyse the average effects between independent experiments.
-Figure 6 is simply transcriptional profiling with no mechanistic investigation. There is no additive reason to include this in this manuscript. It is not clarifying or further investigated.

Minor comments

First revision

<u>Author response to reviewers' comments</u>

We thank the two reviewers for their helpful comments. A point-by-point response is below. We also updated our graphs to address editorial comments on data presentation.

Reviewer 1: This study is a follow up to a detailed study from 2023 in Biophys J by the same group that showed Rho activation triggers eversion of MDCK cell acini to an "apical-out" organization. Notably, they also showed that simple mechanical disruption of the acini by laser ablation was

© 2026. Published by The Company of Biologists under the terms of the Creative Commons Attribution License (https://creativecommons.org/licenses/by/4.0/).

sufficient to trigger eversion, suggesting that Rho activation is not necessary but promotes eversion by creating local breaches in the junctions between the MDCK cells, through increased actomyosin contractility. These data make it a little unclear why they focus on integrin subunits and do not test if knocking out alpha6 integrin reduces these intercellular breaches. They should test the effect of laser ablation on the alpha6 KO cell acini.

Our hypothesis is that eversion may occur through a series of multiple cellular events. The first event appears to be local breaches in cell-cell junctions (noted by the reviewer), as demonstrated by our laser ablation studies in the 2023 Biophysical J paper. However, we hypothesized that integrins may be required to enable cellular migration required to complete the switch from apical-in to apical-out polarity.

Per the reviewer's suggestion, we performed laser ablation experiments with α6 knockout cells, which do not evert following laser ablation (Supplemental Figure 4).

Additionally, we used the Flipper-TR fluorescent cell membrane tension probe to assess if there are any underlying tension differences between wild-type and α6 knockout cells (Supplemental Figure 3). Interestingly, while we observed a slight decrease in both apical and basal membrane tension in α6 knockout cells. However, Rho activator restored membrane tension to wild-type levels in these cells. We therefore conclude that there is not an inherent mechanical defect in these cells that is preventing their response to RhoA.

Overall, the study fills in several details about the phenomenon of acinus eversion to an apical-out configuration, although many of the experiments seem to be unrelated to one another and mechanism underlying the requirement for alpha6 integrin is lacking.

Page 8 - it is unclear why the authors think an antibody (alpha blocking Ab) could penetrate through the tight junctions of the everted MDCK acini to affect the interior cells.

To clarify, α6 blocking antibody and Rho treatment were added simultaneously, which may provide sufficient time for α6 blocking antibody to bind to the basal membrane of cells, thereby preventing eversion. We also have included new images (Supplemental Figure 1) showing the specificity of the blocking antibody (as requested by reviewer 2). In these images we show that simultaneous Rho treatment does not interfere with α6 antibody binding to the basal surface of cells.

The authors note that FAK inhibition reduces infilling of lumens - could this be simply because of reduced cell proliferation? (since they observed reduced infilling when they treated with aphidocolin to inhibit proliferation). They should test this possibility, because if this is the case it is not interesting.

Thank you for this suggestion that FAK inhibition could work to block eversion by limiting cell proliferation. We directly tested the ability of the Y15 FAK inhibitor to alter cell proliferation. However, we did not observe any decrease in proliferation for MDCK cysts treated or not treated with Y15 (Supplemental Figure 5).

There are problems with the RNAseq - the authors ascribe changes in gene expression to eversion but it seems more likely they are caused principally by Rho activation - they have no good control for this and they do not test any of the potential hits. They try using alpha6 knockout cells as a control, but this knockout will have multiple other effects. In addition, their previous paper

© 2026. Published by The Company of Biologists under the terms of the Creative Commons Attribution License (https://creativecommons.org/licenses/by/4.0/).

suggests that Rho activation is not the primary cause of eversion, but works because it damages intercellular junctions.

The authors also do nothing with these RNAseq data and it is unclear why they were included. They are not even mentioned in the Discussion. For these reasons I think that the RNAseq data should be removed from the manuscript.

We agree with the suggestion and have removed the RNAseq data from the manuscript.

Much of the Discussion is related to cancer and reversed polarity, but the treatment of MDCK cells in culture with a potent bacterial toxin to activate RhoA is decidedly unphysiological and the connection to cancer is tenuous at best. Much of this discussion could be removed.
We have limited our discussion of cancer studies to note that there are *in vivo* phenotypes with reversed/inverted polarity that are relevant in the context of cancer.

Discussion - the authors state that "apical polarity is a marker for changes in epithelial cell phenotypes" - but I do not understand what this means. Isn't apical polarization itself a change in phenotype?

We have revised this sentence to state: " Our study presents the concept that inverted (apical-out) polarity may represent a marker for more aggressive epithelial cell phenotypes, including increased proliferation and anchorage-independence. It is an open question if apical-out polarity itself promotes these cellular phenotypes."

Minor and typographical issues:

*The authors should add in the figure legends the meaning of the data points (biological replicates or technical replicates?) and the meaning of the ***s to signify significance (the statement "using *p<0.05 as per ANOVA" is insufficient).*

The *'s used to indicate significance are now detailed in the statistics methods.

page 5, line "Tunnel" 38 should be TUNEL.

Corrected

page 7, line30 "MDCK cells with ("the "with" should be deleted.

This text was revised and corrected.

page 7, line 40 "does not any" - need to add "contain"???

This text was revised and corrected.

Page 8, line 17 "reversion" should be "eversion". Also on line 28.

Thank you for catching this, we have corrected both instances.

© 2026. Published by The Company of Biologists under the terms of the Creative Commons Attribution License (https://creativecommons.org/licenses/by/4.0/).

Page 10, line 31, "feature epithelial cancers" should be "feature of epithelial cancers".

This sentence was removed as part of the reduction of cancer-related topics in the discussion.

Reviewer 2: The manuscript by Patel and colleagues examines the requirement for a6 and b4-integrins in the process of Rho activator-induced eversion of epithelial cysts in 3-dimensional culture. This builds on previous work from the authors and other laboratories understanding how epithelial cells can have alternate configurations of apical-basal polarity orientation.

The manuscript identifies a requirement for a6 and b4-integrins in the process of eversion upon RhoA activator treatment. Notably, the authors also indicate that eversion is accompanied with increased proliferation. This can be largely reverted by aphidicolin treatment to attenuate proliferation or treatment with a FAK inhibitor, providing some molecular insights into the process.

The work is largely descriptive and phenomenological. It contains little mechanistic insight into how these insights between Rho activation, integrin requirement, FAK signalling or proliferation increases are mechanistically linked. It compares Matrigel and Collagen, but these experiments provide no mechanistic advance other than these two matrices being different. Despite these major shortcomings, I think a short, concise and not overclaiming manuscript could be of benefit for the field, if some items are removed from the manuscript, and quantitation provided. I detail these below.

SUGGESTIONS TO AUTHORS

Major comments [Please request additional experiments only if they are essential for supporting the conclusions; authors should be encouraged to highlight any claims that are preliminary or speculative, or to discuss any pitfalls or alternative interpretations in a 'Limitations' section]

-It would be very helpful at the start of the manuscript to help orient the reader by including a cartoon schema of the eversion process from your and others' previous work.

We agree that a cartoon of the process would be helpful. A schematic is included in Figure 6 to summarize our current understanding of eversion (taking into account our work from this study). We chose to present this in the discussion rather than the introduction to account for the findings from this manuscript.

-You describe that "a6 and β4 exhibited lumen collapse following Rho Activator II treatment (Figure 1A, 1B), indicating that lumen collapse is not dependent on these integrins. The quantitation in Figure 1B for b4-integrin KO displays an increase in disrupted lumen category, but whether this is significant is unclear. However, the image in Figure 1A for s6 KO does not seem to show disrupted lumen formation, nor does the quantitation in Figure 1B. Rather, it seems to show resistance to Rho Activator II effects. Therefore, this data is not described correctly. Therefore a6 and b4 appears to have distinct roles.

We appreciate the reviewer’s attention to the differences in α6 and β4 knockout phenotypes. This graph is now relabeled to show that there is an increase in the disrupted lumen phenotype for β4 knockout cysts treated with Rho Activator. Additionally, the results section is updated to emphasize this.

© 2026. Published by The Company of Biologists under the terms of the Creative Commons Attribution License (https://creativecommons.org/licenses/by/4.0/).

-Figure S1 lacks quantitation of phenotypes. All conclusions relating to effects of integrin KO in this figure require statistical support. Since these integrins are not studied further, in lieu of quantitation of these phenotypes, the authors could simply remove Figure S1 and the corresponding main text.

Thank you for this suggestion, we have removed this figure.

-Figure S2 lacks quantitation. Since the authors conclude "a6 blocking antibody had minimal effects on apical-basal polarity and did not disrupt single lumen morphology (Supplemental Figure 2)" the authors need to provide quantitation.

We did not observe any significant instances of disruption of a lumen following α6 blocking. However, this is not a significant claim for this paper we have deleted this image and the statement from the text.

-Figure 1D lacks an antibody isotype control for anti-a6-integrin blocking antibody treatment. Moreover, the specificity of this antibody is not provided, such as lack of effect in a6 KO cells.

We appreciate this suggestion. Please see supplemental figure 1 which shows that the α6 blocking antibody does not bind to α6 knockout cells. It also shows clear basal labeling of wild-type MDCK cysts. We hope these additional images confirm the specificity of the α6 blocking antibody (which has been previously established in the literature as a functional blocking antibody).

-In Figure 2A, you state "spheroids cultured in collagen had a mixed phenotype in which the laminin network was either observed on the basal (outer) side of the spheroid or instead as assembled laminin at the apical surface (Figure 2A)." Please provide quantitation.

We have performed analysis of the lamin staining intensity (based on pixel intensity values) for apical and basal surfaces, which show statistically significant differences between matrigel and collagen conditions. See the new quantitation in Figure 2B.

In Figure 3C, D. Statements are made about the location and level of apoptosis. No quantitation is provided for assessment.

We have added additional graphs to analyze the number of apoptotic cells. See the graphs in Figure 2D and 2F.

-In Figure 4B, please indicate if this represents 3 technical replicates of the same experiment, or 3 independent experiments.

This is 3 independent experiments. We have clarified this and other figures in the figure legends with regards to experimental design and number of samples.

I-n Figure 5B, please indicate if this represents multiple cysts from same experiment or from independent experiments. If independent, then it is appropriate to analyse the average effects between independent experiments.

© 2026. Published by The Company of Biologists under the terms of the Creative Commons Attribution License (https://creativecommons.org/licenses/by/4.0/).

These data represent the percentage of cells proliferating per cyst. 10 cyst per experiment were analyzed, for a total of 30 cysts (3 independent experiments). We have clarified this and other figures in the figure legends with regards to experimental design and number of samples.

-Figure 6 is simply transcriptional profiling with no mechanistic investigation. There is no additive reason to include this in this manuscript. It is not clarifying or further investigated.

These data have been removed.

Second decision letter

MS ID#: jcs.264323R1

MS TITLE: Apical-out polarity in epithelial spheroids requires α6β4 integrins, cell proliferation, and anchorage-independence

AUTHORS: Niharika Patel; Jenna Pirrello; Skylar Lynch; Hailee Patel; Bradley J. Sweeney; Shivam Priya; Tanmay P. Lele; Aki Manninen; Daniel Conway

ARTICLE TYPE: Research Article

Dear Dr Conway,
We have now reached a decision on the above manuscript.

As you will see, one reviewer is fully satisfied and the other raises two issues. The first is a question of how best to present technical replicates across independent experiments- please respond and support your decision if you prefer to maintain the present format. The second is that data should be shown and quantified or not discussed, which I agree with. I hope that you will be able to address these two points because I would like to be able to accept your paper. Please be very clear in your response to reviews as I am hoping to evaluate the response myself without sending to reviewers.

Reviewer 1

SUMMARY OF THE ADVANCE MADE IN THIS PAPER AND ITS POTENTIAL SIGNIFICANCE TO THE FIELD

Same as original submission

SUGGESTIONS TO AUTHORS

The authors have made a serious attempt to address the comments on the initial submission. They have added new data (measuring plasma membrane tension using Flipper-TR, Supp Fig 3; new images in Supp Fig 1; and data on proliferation in Supp Fig 5). They have quantified more of their experimental data and also deleted information that did not seem relevant to the manuscript (RNAseq section) and shortened the Discussion to remove unnecessarily broad links to cancer. Finally, they have fixed several minor and typographical issues.
Overall, therefore, I believe this revised manuscript is now suitable for publication.

Minor comments

Reviewer 2

SUMMARY OF THE ADVANCE MADE IN THIS PAPER AND ITS POTENTIAL SIGNIFICANCE TO THE FIELD

© 2026. Published by The Company of Biologists under the terms of the Creative Commons Attribution License (https://creativecommons.org/licenses/by/4.0/).

The revised manuscript by Patel and colleagues presents as a much-improved manuscript by focusing on a shorter story with more direct connection between sections. There are some remaining issues, which I think the authors should be able to address with simple changes.

SUGGESTIONS TO AUTHORS

Major comments [Please request additional experiments only if they are essential for supporting the conclusions; authors should be encouraged to highlight any claims that are preliminary or speculative, or to discuss any pitfalls or alternative interpretations in a 'Limitations' section]

Statistical handling
Statistical analyses are handled appropriately. Analyses are performed by including all technical replicate datapoints (eg. Cyst measurement) across experimental replicates in a single condition. This should be presented as the average of the mean of each experiment. This needs to be updated for every figure that has this kind of data.

Remove data lacking quantitation
I previously indicated that since the authors did not provide quantitation of reported effects of different integrin KOs on polarity eversion, then they should remove this figure if they don't provide quantitation. It is inappropriate for the authors to have removed the figure and continue to state the results as 'data not shown' in the text. Either add the quantitation to support your assertions or remove your assertions.

Minor comments

Second revision

Author response to reviewers' comments

Reviewer 1:

No comments to respond to.

Reviewer 2:

Statistical handling

Statistical analyses are handled appropriately. Analyses are performed by including all technical replicate datapoints (eg. Cyst measurement) across experimental replicates in a single condition. This should be presented as the average of the mean of each experiment. This needs to be updated for every figure that has this kind of data.

We have added additional details to each figure legend to address this.

Remove data lacking quantitation

© 2026. Published by The Company of Biologists under the terms of the Creative Commons Attribution License (https://creativecommons.org/licenses/by/4.0/).

I previously indicated that since the authors did not provide quantitation of reported effects of different integrin KOs on polarity eversion, then they should remove this figure if they don't provide quantitation. It is inappropriate for the authors to have removed the figure and continue to state the results as 'data not shown' in the text. Either add the quantitation to support your assertions or remove your assertions.

We have removed the other integrin knockout cell lines from the manuscript, and focused exclusively on α6β4 integrins.

Third decision letter

MS ID#: jcs.264323R2

MS Title: Apical-out polarity in epithelial spheroids requires α6β4 integrins, cell proliferation, and anchorage-independence

Authors: Niharika Patel; Jenna Pirrello; Skylar Lynch; Hailee Patel; Bradley J. Sweeney; Shivam Priya; Tanmay P. Lele; Aki Manninen; Daniel Conway

Article Type: Research Article

Dear Dr Conway,

I am happy to tell you that your manuscript has been accepted for publication in Journal of Cell Science, pending standard publication integrity checks.

© 2026. Published by The Company of Biologists under the terms of the Creative Commons Attribution License (https://creativecommons.org/licenses/by/4.0/).
